# Children in Mental Health Crisis: Pediatric Primary Care Providers’ Role in Bridging Treatment Following Higher Levels of Care

**DOI:** 10.1007/s10880-024-10037-1

**Published:** 2024-08-03

**Authors:** Rebecca A. Ferro, Sarah Edwards, Kelly Coble, Mark Riddle, Shauna P. Reinblatt, Chelsie Ader, Meghan Crosby Budinger, Amie F. Bettencourt

**Affiliations:** 1 Department of Psychiatry and Behavioral Sciences, School of Medicine, Johns Hopkins University, 550 N Broadway, Room 907, Baltimore, MD 21205 USA; 2 School of Medicine, University of Maryland, 737 W Lombard St, Baltimore, MD 21201 USA

**Keywords:** Pediatric mental health, Mental health crisis, Child psychiatry access, Primary care

## Abstract

The current study examines the role of pediatric PCPs in bridging treatment for youth who have experienced mental health crises and the characteristics of these patients for whom PCPs sought psychiatric consultation and referral support from a child psychiatry access program, Maryland Behavioral Health Integration in Pediatric Primary Care. Psychiatric consultation and referral calls between 2012 and 2021 were included if a) the patient was recently seen in a higher level of care and b) the PCP was bridging treatment following the patient’s discharge; 208 calls met criteria. The most common mental health concerns included depressed mood, suicidal thoughts/gestures, and anxiety. Acute concerns of aggression, suicide attempts, and hallucinations were also reported. Over half of the patients had two or more mental health diagnoses. At the time of the call, only one quarter of these patients had outpatient therapy services while about half were receiving medication treatment. Most of these patients were discharged from the higher level of care without a care plan. Pediatric PCPs are managing their patients’ complex mental health concerns following receipt of higher levels of care. Improvements in collaboration and care coordination between pediatric PCPs and emergency department providers are needed.

An estimated one in five children in the United States have a mental health diagnosis, however, only 20% receive necessary treatment (Center for Behavioral Health Statistics and Quality, 2016). When treatment is delayed, or not able to be accessed, mental health crises can develop. Currently, an average of approximately 6000 children (ages 12–17) present to emergency departments (EDs) with a mental health crisis each week in the U.S (Anderson et al., 2023). Between 2011 and 2020, pediatric ED visits for mental health concerns doubled while suicide-related visits increased 5-fold (Bommersbach et al., 2023). The period of time following discharge from a higher level of care (e.g., ED, inpatient, residential facility) is a vulnerable time for children and teens, with increased risk of readmission (Feng et al., 2017). The quality of the discharge plan, including timely follow-up with an outpatient provider, predicts lower rates of return ED visits (Hoffmann et al., 2023). Post-discharge bridging services can reduce ED mental health visits and lower hospital readmission rates for children and adolescents (Simmons et al., 2023). Further, youth who receive a mental health follow-up visit within 7 days of psychiatric discharge have significantly lower odds of suicide during the next 6 months (Fontanella et al., 2020). However, adequate discharge planning appears to be lacking. In a large sample of Medicaid-enrolled children discharged from the ED for a psychiatric complaint, fewer than one-third had outpatient mental health follow-up within 7 days of discharge and only 56% had follow-up within 30 days (Hoffmann et al., 2023).

Following discharge, pediatric primary care providers (PCP) are often the first point of contact for youth. Due to the ongoing shortage of child and adolescent psychiatrists, pediatric PCPs often assume responsibility for addressing their patients’ mental health needs (Anderson et al., 2015; Olfson et al., 2014). However, PCPs have reported feeling unprepared to manage the often complex mental health needs their patients present with in the primary care setting (Osborn et al., 2015). Models of integrating behavioral health with primary care are one approach to address issues in access to and continuity of care. These models exist on a continuum from consultation and collaboration between medical, psychology and psychiatry professionals to co-location and complete integration (Njoroge et al., 2016). For a meta-analysis on efficacy of integrated care models see Asarnow et al., 2015.

Across the U.S., 47 child psychiatry access programs (CPAPs) have been established to provide support to pediatric PCPs in addressing the mental health needs of their patients and improve coordinated care efforts (National Network of Child Psychiatry Access Programs, 2023). Maryland Behavioral Health Integration in Pediatric Primary Care (BHIPP) is one such CPAP that was formed in 2012. BHIPP operates as a partnership among several universities across the state of Maryland and provides telephone consultation, resource and referral networking, training, and direct services including telemental health evaluation and care coordination and direct-to-patient mental health intervention by social work interns co-located in select practices. During the provision of clinical consultation services, the BHIPP team observed that PCPs were often calling for assistance as they bridged treatment from a higher level of care. A few calls to the BHIPP line will be illustrated to demonstrate the level of need and nature of the cases that PCPs are managing.

## Case Illustrations

Increasingly, pediatric PCPs request BHIPP consultation for complex cases that require a multi-pronged response including guidance around medication and treatment approaches, and resource and referral assistance. In one such instance, a PCP contacted BHIPP for a consultation regarding a 10-year-old female with an extensive psychiatric history including a diagnosis of bipolar disorder at age 4. The patient’s treatment history included several unsuccessful medication trials with selective serotonin reuptake inhibitors and antipsychotic medications, and visits to an ED. At the time of the call to BHIPP, she was not receiving therapy or medication treatment. The child’s PCP reported a recent decompensation with worsening emotional and behavioral dysregulation, aggression, and threatening behavior in multiple settings, including school, home and the pediatric practice. In a recent incident, after her mother set a limit, the patient unbuckled her seatbelt and began clawing at her mother's face while she was driving. Following this, the patient was brought to her local ED. However, finding an appropriate placement was challenging and the patient was discharged without an identified placement. The BHIPP Consultant provided the PCP with specific medication recommendations to stabilize the patient and address the patient's impulsive and dysregulated behavior while awaiting placement in an acute care setting. The PCP was encouraged to call BHIPP back as needed while the patient was being stabilized and was given instructions on what to do if symptoms worsened. In addition to treatment guidance, the BHIPP team was able to provide the patient with appropriate referrals for specialized acute care and outpatient psychotherapy as well as psychoeducational handouts to assist this PCP with explaining the nature and course of the patient’s condition to the family.

Some calls to the BHIPP consultation line are solely requests for resources and referrals, particularly when patients are discharged with no or inadequate discharge plans. One such provider called regarding a 13-years-old female who had been recently discharged from the hospital following a suicide attempt with no follow-up in place. BHIPP was able to provide several community-based outpatient psychotherapy referrals that accepted the patient’s insurance and had short wait times for an appointment.

BHIPP also provides guidance to PCPs on navigating barriers to care, such as insurance coverage. A PCP called about her 16-year-old patient with anxiety and anorexia nervosa. The patient had a history of three inpatient admissions for an eating disorder, however she had been repeatedly discharged early due to issues with insurance coverage. The PCP had contacted the insurer to obtain long term inpatient treatment options, but the referrals that were sent were not clinically appropriate. The PCP was seeing the teen weekly in addition to an outpatient therapist and psychiatric nurse practitioner, yet the patient’s disordered eating behaviors were worsening and a higher level of care was indicated. The BHIPP Consultant advised the PCP about a walk-in mental health clinic and an additional intensive treatment facility that accepted the patient’s insurance. Additionally, the BHIPP Consultant provided concrete steps and information regarding patient advocates, targeted case management, single-case agreements, and voluntary placement.

BHIPP consultants are also able to provide clinical guidance to PCPs for children experiencing a mental health crisis when other care is not easily accessible. A PCP called for a consultation about a 10-year-old male with symptoms of depression, anxiety, and attention-deficit/hyperactivity disorder (ADHD) who had been making suicidal statements. The patient had been stable on medications for two years but had a recent dosage adjustment and a disruption in care due to his therapist being on extended leave. The patient presented to an ED following a suicide attempt, but was released after approximately 8 hours without an evaluation by a mental health professional because he was no longer expressing suicidal ideation. In turn, the family presented to the child’s PCP for treatment and guidance as they were now hesitant to return to the ED. The BHIPP Consultant was able to provide real-time support to the child’s PCP in conducting safety planning, prescribing medication treatment, and recommendations and referral assistance for outpatient psychotherapy and psychiatry.

## Current Study

The current study examined characteristics of consultation and referral calls for pediatric patients stepping down from an ED or other higher level of care (e.g., inpatient, residential treatment) and investigated how pediatric PCPs bridge treatment for these patients, provide continuity of care for and reduce gaps in treatment. The study aims to illustrate the role of pediatric PCPs in addressing mental health crises to inform systematic changes and opportunities for further support following treatment in higher levels of care.

## Methods

BHIPP provides two types of telephone-based services, 1) psychiatric consultation and 2) resource/referral networking calls. The BHIPP consultation line is staffed by master’s level behavioral health consultants who triage calls–either providing resources immediately or connecting the pediatric provider with a child and adolescent psychiatrist for consultation. This study is a secondary analysis of programmatic data collected during the provision of BHIPP services from the date of program inception in October 2012 to December 2021. BHIPP calls that pertained to patients with prior or current treatment in a higher level of care (i.e., ED, inpatient psychiatric facility, or residential treatment setting) were pulled for further analysis (*n* = 254).

All study authors, who are trained mental health professionals, were randomly assigned and reviewed call data to determine if the cases were appropriate for inclusion in the study. Cases were included if any of the following criteria were met: 1) the PCP managed a patient following receipt of higher level of care 2) the PCP followed up with the family at a higher frequency than is typical after the patients’ receipt of higher level of care 3) the PCP served a care coordination role following a higher level of care. After review of cases for bridging treatment criteria and team discussion, the following additional higher levels of care were added: child abuse centers (*n* = 1) and a local walk-in crisis clinic (*n* = 3). Cases were included even if the presenting concern was primarily about the parent’s mental health (i.e., factitious disorder). Cases were excluded if the higher level of care occurred more than 3 months prior to the BHIPP call. Calls that pertained to PCPs requesting to transition a patient between facilities that were of a similar higher level of care without seeing the patient in the interim were not considered bridging treatment and therefore not included in the study (e.g. moving a patient from one inpatient facility to another). Inter-rater reliability was conducted to ensure that calls identified by authors were consistently meeting the bridging treatment criteria. A total of 20% (*n* = 50) of the cases were coded by two authors with an overall reliability rate of 86%. After exclusion criteria, the final sample consisted of 208 calls (129 consultations and 79 referrals).

During all calls, BHIPP staff collected de-identified data on patient demographics (e.g., gender, race/ethnicity, age), insurance type (e.g., private, public), presenting concerns (e.g., suicidal ideation or attempt, aggression, etc.), diagnostic impression, adverse childhood experiences and current and prior behavioral health treatment received. Information on missing and unknown data is reported in the Results and Tables. For consultation calls, extensive notes were taken regarding the nature of the call and other pertinent clinical information (e.g., patient was discharged from the ED without a care plan in place). Additionally, BHIPP child and adolescent psychiatrists rated patient severity with the Clinical Global Impression Score (CGI-S) immediately following each consultation call. The CGI-S is rated on a 7-point scale: 1 = *normal, not at all ill*, 2 = *borderline mentally ill*, 3 = *mildly ill*, 4 = *moderately ill*, 5 = *markedly ill*, 6 = *severely ill*, 7 = *among the most extremely ill patients* (Guy, 2000). Research Electronic Data Capture (REDCap) tools hosted at Johns Hopkins University were used to collect and store data (Harris et al., 2009, 2019). IRB approval was obtained from Johns Hopkins University, University of Maryland, and Maryland’s Department of Health.

### Statistical Analyses

Descriptive statistics and chi-square analyses were used to examine the role of pediatric providers, patient characteristics, the differences between patients who were the subject of psychiatric consultation calls versus referral calls and the differences between patients with higher severity and lower severity ratings.

## Results

The majority of calls (92.3%) pertained to pediatric patients who were in a higher level of care (ED, inpatient or residential) for a psychiatric emergency within the past three months while 7.7% were regarding patients currently in a higher level of care. Overall, 63.0% of patients received services in the ED, 43.3% were treated at an inpatient facility and 2.4% received residential services. During the data collection period (October 2012–December 2021) the mean number of calls per year was 20.8 (SD = 13.71) with the most calls (*n* = 43; 20.7%) received in 2021. Nineteen of the patient-specific calls concerned the same patients, this data was excluded from demographic analyses to avoid duplication; however, all other analyses included these calls. Patients were on average 14.06 years old (SD = 3.83), majority female (58.2%) and White (48.1%) or African American (18.5%); see Table 1 for full demographics. Only one call primarily concerned the caregiver’s mental health in a case of suspected factitious disorder.

**Table 1:** Patient demographics

Demographics	*N*	%
Gender		
Female	110	58.2
Male	76	40.2
Unknown	3	1.6
Age		
0 to 5	3	1.6
6 to 12	60	31.7
13 to 18	102	54.0
19 and up	23	12.2
Unknown	1	0.5
Race/ethnicity		
White	91	48.1
African American	35	18.5
Asian	5	2.6
Latino	10	5.3
Other	4	2.1
Unknown	54	28.6
Insurance type		
Public only	72	38.1
Private only	87	46.0
Public and private	2	1.1
None/unknown	28	13.5
Urban vs rural		
Rural/semi-rural	63	33.3
Urban/suburban	126	66.7
Total	189	100

19 duplicate contacts removed from demographics

Total sample for this table is *n* = 189

### Pediatric Primary Care Providers’ Bridging Treatment Role

Nearly all of the pediatric PCPs were seeing their patients at a greater frequency, and outside of well child check appointments, following admission to a higher level of care (95.7%). Their roles in bridging treatment included care coordination (92.7%) and other responsibilities such as medication management (54.3%). Additionally, a majority of patients in this sample were discharged from a higher level of care without a care plan in place (66.8%).

### Pediatric Patient Characteristics

Overall, patients endorsed on average 2.6 presenting problems (SD = 1.60) with the most common being depressed mood, suicidal thoughts/gestures, and anxiety. Many patients were reported to have high acuity concerns including aggression, suicide attempts and hallucinations. Approximately half of PCPs (*n* = 111) provided information on adverse childhood experiences (ACEs) in their patients. Of these, 25.5% of patients were reported to have some type of ACE (e.g., child maltreatment, separation from caregiver, loss of a loved one). The most common diagnostic impressions were major depressive disorder, an anxiety disorder, and ADHD. See Table 2 for more information on presenting problems and diagnostic impressions. Approximately half (52.9%) of the patients had comorbid psychiatric diagnoses. Table 3 contains information on patient severity, comorbidity, medication type and polypharmacy.

**Table 2:** Patient presenting problems and diagnoses

Presenting problem	*N*	%
Depressed mood	86	41.3
Suicidal thoughts/gestures	77	37.0
Anxiety	62	29.8
Behavior problems at home	39	18.8
Aggression	31	14.9
Eating/feeding problems	18	8.7
Suicide attempt	18	8.7
Behavior problems at school	17	8.2
Substance use	17	8.2
Hallucinations	16	7.7
Parent-child conflict	15	7.2
Impulsive behaviors	14	6.7
Cutting/self-injury	11	5.3
Diagnosis		
Major Depressive Disorder	97	46.6
Anxiety Disorder	76	36.5
ADHD	39	18.8
Mood Disorder	16	7.7
Bipolar Disorder	13	6.3
Disruptive Disorder/ODD	23	11.1
Trauma or Stressor Related	19	9.1
Autism	18	8.7
Psychotic Disorder	17	8.2
Eating Disorder	15	7.2
Substance Use Disorder	12	5.8
Developmental Disorder	5	2.4
Dysthymia	4	1.9
Adjustment Disorder	3	1.4
Comorbid Medical	3	1.4
Learning Disorder	2	1.0
Other	24	11.5
Unknown or N/A	37	17.8

Only presenting concerns endorsed by 5% or more of the total sample are included in the table, additional categories were: adjustment, anger/irritability, attention/concentration, avoidance, compulsive behavior, delusions, destructive behavior, developmental delay/concerns, dissociation, elimination problems, elopement, emotional dysregulation, expansive mood, family discord, grief, homicidal thoughts/gesture, hurting animals, hyperactivity, labile mood, legal problems, medical concern, medication side effects, obsessive thoughts, pseudoseizures, reckless/risky behavior, school refusal, self-esteem/body image; sexual acting out, sexual/gender identity, sleep problems; somatic complaints, tics (motor/vocal), truancy, underachievement at school, and worries/fears.

**Table 3:** Patient comorbidity and medication use

Comorbidity and medication	*N*	%
Severity		
Moderately ill (4)	46	35.7
Markedly ill (5)	54	41.9
Severely to extremely ill (6-7)	26	20.2
Missing	3	2.3
Comorbidity		
No diagnosis	11	5.3
1 diagnosis	87	41.8
2 diagnoses	61	29.3
3 diagnoses	31	14.9
4 or more diagnoses	18	8.7
Medication type		
Antidepressants	58	27.9
Antipsychotic	39	18.8
ADHD	33	15.9
Anxiolytics	17	8.2
Mood stabilizer	15	7.2
Sleep aid	3	1.4
Other	2	1.0
Polypharmacy		
No medication	94	45.2
1	68	32.7
2	24	11.5
3 or more	22	10.6

Severity scores were only available for consultation calls (*N* = 129)

The medication and polypharmacy data here represents current medication use

At the time of the BHIPP call, 62.5% of patients were currently receiving some type of outpatient mental health services aside from a higher level of care, including outpatient therapy (28.8%) and medication treatment (51.0%). Medications were most often prescribed by PCPs (72.3%), followed by a mental health provider (23.4%) or both (4.3%). The most commonly prescribed medications were antidepressants, antipsychotics and ADHD medications. Nearly a quarter (22.1%) of patients were prescribed more than one medication. Previous mental health treatment aside from a higher level of care was much less common, with only 23.6% reporting having received services, the majority of which received medication treatment. Past medication use appeared to be less frequent than current medication use, with only 21.6% of the patients having a prior history of being prescribed psychotropic medication. However, the most commonly prescribed prior medications were consistent with current medication use–antidepressants (11.1%), ADHD medications (7.2%), and antipsychotics (6.7%).

The primary recommendations from BHIPP staff following the call were referral to mental health or community resources (75.0%) and medication evaluation or change (42.8%). Approximately 15.8% of patients were recommended to return to a higher level of care (ED: 9.1%, inpatient: 5.8%, or residential: 1.0%) and 12.9% were advised to seek intensive outpatient services (day hospital: 6.3% and intensive outpatient: 6.7%).

### Comparison of Patient Characteristics by Service Type

BHIPP calls requesting referral services were more often from urban or suburban primary care practices (88.6%), compared to consultation calls (49.6%), *χ*^2^(1) = 32.51, *p *< .001. There was no statistically significant difference in the number of presenting concerns endorsed by service type, *χ*^2^(9) = 9.71, *p *= .375. However, consultation calls more often pertained to concerns of anxiety (34.9% consultation; 21.5% referral), *χ*^2^(1) = 4.18, *p *= .041. Comorbidity was also higher for consultation calls (62.0%) compared to referral calls (38.0%), *χ*^2^(1) = 11.37, *p *< .001.

### Comparison of Patient Characteristics by Severity Rating

This section explores the differences in patient characteristics by patient severity levels as rated by BHIPP child and adolescent psychiatrists for consultation calls. Approximately 36% were rated as moderately ill, 43.9% as markedly ill, and 20.2% severely-extremely ill. The number of presenting concerns was not significantly different for patients with different severity ratings, *χ*^2^(18) = 22.88, *p *= .195. However, there were differences in the type of concerns. Patients considered to be higher severity more often presented with aggression, and behavior problems at school (CGI-S ≥6: 30.8%), *χ*^2^(2) = 11.69, *p *= .003, compared to those considered less severe. Patients with higher severity were less often diagnosed with an anxiety disorder or major depressive disorder, compared to those with lower severity. There was no statistically significant difference in whether the patient had comorbid psychiatric diagnoses based on patient severity. Similarly, there was no significant difference in current or past receipt of mental health treatment by severity rating. This difference remained non-significant when examining treatment types by level of care (e.g., outpatient, intensive inpatient). However, patients with higher severity were more often prescribed antipsychotic medications, compared to those with lower severity ratings. Those with severity ratings of markedly ill were more often prescribed anxiolytic medications, compared to those of lower or the highest severity. There were no statistically significant differences in polypharmacy by patient severity rating. For additional information comparing patient characteristics by severity rating see Table 4.

**Table 4:** Comparison of patient characteristics by severity rating

	CGI 4		CGI 5		CGI 6-7		Chi-square value
	*N*	%	*N*	%	*N*	%	
Severity rating	46	36.5	54	43.9	26	20.6	
Comorbidity							0.50
No comorbidity	19	41.3	19	35.2	9	34.6	
2+ diagnoses	27	58.7	35	64.8	17	65.4	
Current treatment							0.96
None	15	32.6	13	24.1	8	30.8	
Outpatient	31	67.4	41	75.9	18	69.2	
Day hospital or intensive outpatient	–	–	–	–	–	–	
Past treatment							3.41
None	37	80.4	38	71.7	17	65.4	
Outpatient	9	19.6	14	26.4	9	34.6	
Day hospital or intensive outpatient	–	–	1	1.9	–	–	
Medication							
ADHD	9	19.6	10	18.5	5	19.2	0.02
Antidepressants	21	45.7	15	27.8	8	30.8	3.74
Antipsychotic	4	8.7	10	18.5	14	53.8	20.34***
Anxiolytics	1	2.2	11	20.4	4	15.4	7.63*
Mood stabilizer	1	2.2	6	11.1	4	15.4	4.31
Polypharmacy							
More than 1 medication	9	19.6	17	31.5	10	38.5	3.30
Presenting problem							
Aggression	3	6.5	11	20.4	9	34.6	9.07**
Behavior problems at school	2	4.3	5	9.3	8	30.8	11.69**
Diagnosis							
Anxiety disorder	25	54.3	29	53.7	6	23.1	7.92*
Major depressive disorder	30	65.2	21	38.9	10	38.5	8.19*

A CGI-S rating was missing for 3 consultation calls

Current and past treatment do not include higher levels of care (ED, inpatient, residential)

Only significant differences in presenting problems and diagnoses are presented

CGI-S data was only available for consultation calls (*N* = 129)

“–” indicates that patients were not receiving treatment in a day hospital or intensive outpatient program

***p</=.001

**p</=.01

*p</=.05

## Discussion

There is a paucity of research describing pediatric PCPs involvement after youth receive intensive mental health evaluation and treatment despite the growing trend for youth to present to EDs for mental health concerns (Nadler et al., 2021; Sheridan et al., 2021). Our findings highlight the crucial role pediatric PCPs play in helping youth transition from higher levels of psychiatric care. In this study, PCPs reported providing care and support for youth with complex mental health needs. Over half of the pediatric patients in this sample were diagnosed with multiple mental health disorders. Primary presenting problems included depressed mood, suicidal thoughts and gestures, and anxiety in addition to higher acuity concerns such as aggression, suicide attempts, substance use, hallucinations, and self-injury. The comorbid nature of these concerns likely contributed to the severity of presentations, thus necessitating higher levels of care. Approximately half of these pediatric patients were taking psychiatric medications, but only a quarter had outpatient therapy services in place at the time of their discharge from the higher level of care. Moreover, almost two-thirds of these patients were discharged from higher levels of care without a care plan in place.

These findings are concerning given the critical importance of promoting continuity of care following a mental health crisis. PCPs are bridging treatment gaps and were intensely involved in the support of these patients via medication management, resource connection and frequent follow-up appointments. In fact, nearly all of the pediatric PCPs saw these patients at increased frequency, with over 90% also helping to coordinate care. Given the frequent lack of a care plan upon discharge, it is also not surprising that up to 16% of our sample were recommended by BHIPP to return to a higher level of care, (e.g., ED or inpatient unit) and 13% to seek intensive outpatient services (e.g., day hospital or intensive outpatient program). Specific adjustments to discharge planning procedures could be considered, especially for high-risk presenting concerns (suicidality, aggression, psychosis) that are likely to lead to poor outcomes and re-admission.

Our results support literature describing challenges ensuring appropriate follow-up care for children and adolescents discharging from acute care settings. Lynch and colleagues (2021) found fewer than half of youth seen in the ED for mental health concerns received care coordination as defined as a follow-up visit with PCP or specialty care (Lynch et al., 2021). They hypothesized that workforce shortages, long wait times for appointments, and lack of reimbursement for care coordination are key contributors to low rates of care coordination and follow-up care. The implications of insufficient follow-up care include repeated illness exacerbations, requiring higher levels of care to target recalcitrant mental illness and increased utilization of health care services that may increase wait times and lower accessibility to such health care (Hoffmann et al., 2023; Simmons et al., 2023). Another complication here is whether visits to EDs are clinically necessary. Trends in ED visits for mental health crises in pediatric patients suggest a rise in urgent and non-urgent concerns (Hoge et al., 2022). Patients in this study were primarily rated as moderately or markedly ill at the time of BHIPP contact and most had multiple presenting concerns; however, it is possible that the acuity for some concerns may not have necessitated an ED visit. Lack of access to other supports, including primary care, could also result in increased use of EDs.

While barriers to accessing outpatient mental health care certainly do exist, a recent quality improvement study found a multifaceted intervention using electronic medical record alerts and provider education enhanced communication and PCP follow-up for adolescents presenting to the ED for a nonpsychiatric complaint but screening positive for depression or suicide risk (Esposito et al., 2020). It is recommended that CPAPs serve as a partner by providing education and training on the importance of and strategies for increased communication and care coordination between the ED team and PCP. Additionally, providing training jointly to ED providers and PCPs on topics related to medication management may increase ED provider’s confidence in starting medication if they know the PCP could effectively manage the patient or bridge the patient until the connection to a psychiatric provider is made. The need for adequate discharge planning has been identified; however, this issue is complicated by workforce shortages, access issues and billing/reimbursement challenges. Simply requiring that primary care or specialty mental health appointments be established as part of discharge planning may lead to increases in other problems such as boarding mental health patients in the ED. A multipronged approach that includes advocacy for bolstering specialty mental health services, including mobile crisis, and improvement in reimbursement for care coordination may help to reduce challenges faced by EDs and PCPs alike in addressing pediatric mental health. CPAPs can support continuity of care by maintaining databases of local mental health services with information regarding accepted insurance types and wait times for treatment. CPAPs can also establish relationships with EDs to assist in direct care coordination with families.

### Limitations

While the current study provides additional data on pediatric mental health crises and primary care, there are limitations to consider. The data were from a single CPAP in Maryland and may not be generalizable. This study was a secondary analysis of BHIPP program data which was not originally designed to capture bridging of care and did not include data from medical records. The BHIPP database is limited to the information collected by the PCP and in turn reported to BHIPP as opposed to being directly collected from patients/families. This method of data collection could result in a filtering of information (e.g., complete and accurate treatment history, previous prescribers) whereby all information may not be relayed to the PCP or BHIPP. The measure of severity used in this study (CGI-S) is widely used in psychiatric research and relies on clinical judgement of a patients’ functioning; yet, it lacks specific behavioral anchors and may be subject to rater bias. Lastly, the coders were not blinded to the study purpose and while there was oversight to ensure inter-rater accuracy and consistency, all database reviews are susceptible to the potential for coding inconsistencies and errors.

## Conclusion

The current study suggests that pediatric PCPs who call CPAPs are managing the complex mental health needs of their pediatric patients following the patients’ stays in higher levels of care. The findings illustrate the multiple roles pediatric PCPs play in bridging care including increased follow-up visits, medication management and care coordination. Our findings have important implications for the role of CPAPs in supporting pediatric PCPs caring for their patients following higher levels of care, particularly through providing training and education in mental health practices. However, CPAPs may also contribute by promoting communication between EDs and PCPs through assisting with patient discharge planning. Further studies are needed to examine how to improve care coordination from higher levels of mental health care prior to the patient’s return to their PCP. Initiatives to enhance collaboration between higher levels of care and primary care using CPAPs are indicated. Future research should assess the impact of CPAP efforts to promote continuity of care.

## Data Availability

This data is not available for public use.
